# Advanced Polybenzoxazine Structures Based on Modified Reduced Graphene Oxide

**DOI:** 10.3390/polym10090941

**Published:** 2018-08-24

**Authors:** Elena Iuliana Bîru, Sorina Alexandra Gârea, Alina Nicolescu, Eugeniu Vasile, Horia Iovu

**Affiliations:** 1 Advanced Polymer Materials Group, University Politehnica of Bucharest, 011061 Bucharest, Romania; iuliana.biru@upb.ro (E.I.B.); garea_alexandra@yahoo.co.uk (S.A.G.); 2 Petru Poni Institute of Macromolecular Chemistry, 700487 Iasi, Romania; alina@icmpp.ro; 3 Department of Oxide Materials Science and Engineering, University Politehnica of Bucharest, 011061 Bucharest, Romania; eugeniuvasile@yahoo.com; 4 Academy of Romanian Scientists, 050094 Bucharest, Romania

**Keywords:** modified graphene oxide, polybenzoxazine, exfoliated graphene sheets

## Abstract

Two new polybenzoxazine products based on a reduced graphene oxide (rGO) were synthesized. The synthesis process started with an rGO with amino functionalities. Benzoxazine structures were synthesized with oxazine rings attached to the surface of rGO and were fully characterized by FT-IR, ^1^H–NMR, XPS and XRD. The presence of polybenzoxazine chains was pointed out by ^1^H–NMR and it correlated with XRD data which show a partial exfoliation of the graphene oxide layers. The degree of polymerization plays a significant role against the exfoliation process. A higher yield of the ring-opening process for benzoxazine rings leads to a low degree of exfoliation, as the inner covalent bonds within the polybenzoxazine chains keep the graphene oxide sheets together.

## 1. Introduction

Strong efforts have been made, in the last decade, to get high-performance polymer-based composites to meet recent demands in the electronic industry. Due to their outstanding properties, polybenzoxazine resins have been extensively studied for many applications, such as for electrochromic materials [1], removal of mercury salts [2], gas separation membranes [3,4,5], and electro-catalysts for water splitting [6]. When synthesized by the condensation reaction of inexpensive raw materials, such as primary amines, phenols and formaldehyde, the benzoxazine monomers can be easily polymerized [7] through the ring-opening mechanism, without adding any catalyst. Because of its extensive H-bonding networks [8], the polybenzoxazine (PBZ) resins exhibit molecular-design flexibility, low water absorptions, high glass transition temperature (*T*_g_), near-zero volumetric shrinkage upon curing, and low flammability [9]. Despite these important properties, the applications of pure-based benzoxazine polymers in high-tech industries have been limited, due to their brittleness, high curing-temperature and difficulty in processing them—especially into thin films and coatings [10].

In 2004 [11] graphene was isolated as a two-dimensional layer composed of sp^2^ carbon atoms arranged in a honeycomb crystal structure. Since then, it continues to attract considerable interest because of its large applications as energy storage systems [12], batteries and supercapacitors [13], drug delivery systems [14], nanomaterials for removal of heavy metals [15], etc. Graphene sheets can be easily folded without causing them to break and they conduct heat and electricity better than any metal. However, graphene has a low dispersibility in common solvents which is a hindrance to its applications and functionalities [16]. As a substitute, graphene oxide is a promising candidate for making new polymer composites with enhanced properties, as it can establish covalent bonds between the monomer and its numerous oxidized groups. Recent studies have shown that incorporating graphene oxide in the polybenzoxazine mass significantly improves the polymer`s electrical properties [17], as well as its corrosion resistance, due to the barrier properties of graphene oxide [18].

We have previously reported the successful growth of the benzoxazine monomer on the surface of carboxylated graphene oxide, using two different methods, and have shown that the benzoxazine polymerization may occur either between the rings of the same graphene oxide (GO) sheet (“in-graphene polymerization”) or between the rings of different GO sheets (“out-graphene polymerization”) [19]. However, the abundant presence of oxygen functional groups causes significant damage to the sp^2^ continuous network of graphene and lowers its electrical properties [20]. As a result of the reduction of GO, the π–π (sp^2^) network is in great measure re-established as compared to GO. Additionally, the residual oxygen functionalities present in rGO allow for better dispersibility in an organic solvent [21].

Here, two different types of aminated reduced graphene oxide were used in order to form the covalent benzoxazine monomer directly on the rGO surface. The aminated rGO was used in this study as a primary amine component in the Mannich condensation reaction, along with phenol and formaldehyde. This lead to new hybrid rGO-benzoxazine based polymers with exfoliated structure.

## 2. Materials and Methods

### 2.1. Materials

Two types of amino reduced graphene oxide were used in this study and each of them contained a known amount of amino functionalities. They were, namely, covalently linked reduced graphene oxide amino-polyethylene glycol (rGO–NH_2_), and reduced graphene oxide-tetraethylene pentamine (rGO–TEPA) purchased from NanoInnova Technologies (Madrid, Spain). Phenol (≥99%), paraformaldehyde (95%), sodium hydroxide (≥98%), p-xylene anhydrous (≥99%) and hexane (95%) were supplied by Sigma-Aldrich (Taufkirchen, Germany). All reagents and solvents were used as received without further treatment.

### 2.2. Characterization

Fourier Transform Infrared Spectroscopy (FTIR) spectra were registered on a BRUKER VERTEX 70 spectrometer (Bruker, Billerica, MA, USA), in 400–4000 cm^−1^ region. The samples were mixed with KBr powder and pressed in a pellet. The resolution was 4 cm^−1^ and 32 scans were performed. X-ray photoelectron spectroscopy (XPS) analyses were recorded using a monochromatic Al Kα source (1486.6 eV), at a pressure of 2 × 10−9 mbar, on a K-Alpha instrument from Thermo Scientific (Ho Chi Minh, Vietnam). Charging effects were compensated by a flood gun and binding energy was calibrated by placing the C 1s peak at 284.8 eV as an internal standard. Deconvolution of C 1s peaks was done after subtraction of Shirley background. The NMR spectra were recorded on a Bruker Avance NEO spectrometer (Bruker, Rheinstetten, Germany) operating at 400.1 MHz for ^1^H. For the NMR analysis, a 5 mm multinuclear inverse detection z-gradient probe was used. ^1^H–NMR spectra were recorded using a standard pulse sequence, as delivered by Bruker, with TopSpin 4.0.3 spectrometer control and processing software. Thermogravimetric analyses (TGA) were performed with a Q500 TA instrument (TA Instruments, NY, USA) from 30 to 800 °C using nitrogen (gas flow rate of 90 mL/min and 10 °C/min as heating rate). Differential Scanning Calorimetry (DSC) tests were performed on DSC 402 F1 equipment from Netzsch (Selb, Germany). The non-isothermal method was used to scan the samples under nitrogen, from 20 to 300 °C using a 10 °C/min heating rate. The Raman measurements were performed with a Renishaw inVia Raman microscope system (473 nm laser excitation, Renishaw, Brno-Černovic, Czech Republic), using 100× objective and 0.4 mW incident power. The X-ray Diffraction Analyses (XRD) were performed on a Panalytical X’PERT MPD X-ray Diffractometer (Malvern Panalytical, Royston, UK), in the range 2*θ* = 2–60°. An X-ray beam characteristic to Cu Kα radiation was used (λ = 1.5418 Å). Transmission electron microscopy (TEM) images were recorded using FEI Tecnai F30 G^2^STWIN HR-TEM microscope (Hillsboro, OR, USA) working at 300 kV. Samples were dispersed in ethanol by ultrasonication and deposited on a TEM copper grid covered with a thin amorphous carbon film with holes.

### 2.3. Synthesis of Benzoxazine-Functionalized rGO Monomers (rGO–BZ)

**Scheme 1:**
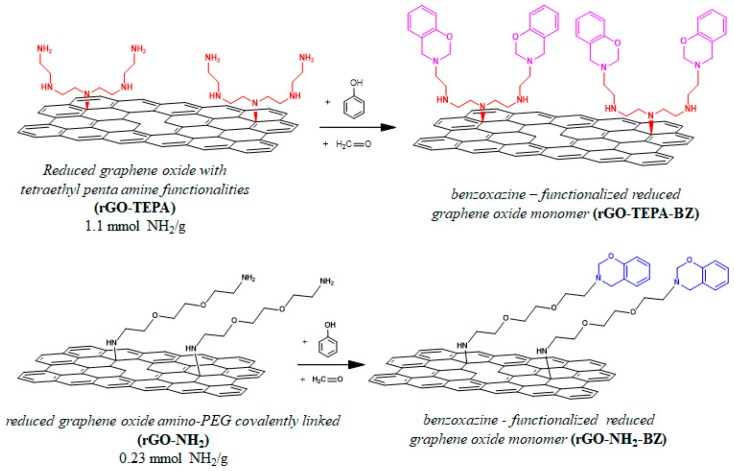
Synthesis of benzoxazine-functionalized rGO monomers.

The synthesis routes of benzoxazine rings covalently functionalized on the aminated rGO surface are shown in Scheme 1. First, the 100 mg of rGO with amino functionalities were dispersed in 30 mL of p-xylene, for 30 min under ultrasonication, keeping the temperature below 10 °C. The necessary amounts of phenol and paraformaldehyde were calculated considering the number of amino functionalities of each rGO type. The benzoxazine hybrid monomers were formed by the condensation reaction of the amine component (rGO–TEPA, and rGO–NH_2_ respectively) with paraformaldehyde and phenol in a 1:2:1 molar ratio. The reaction temperature was increased to 140 °C and maintained under reflux for 6 h. Afterward, the reaction mixtures were cooled to room temperature and the solid products were washed with hexane several times. In order to purify the benzoxazine-functionalized rGO monomers, the synthesized products were precipitated in 1 N aqueous solution of NaOH to remove the unreacted phenol and washed with distilled water several times. Finally, the hybrid monomers were dried under vacuum at 50 °C for 24 h.

## 3. Results and Discussion

**Figure 1:**
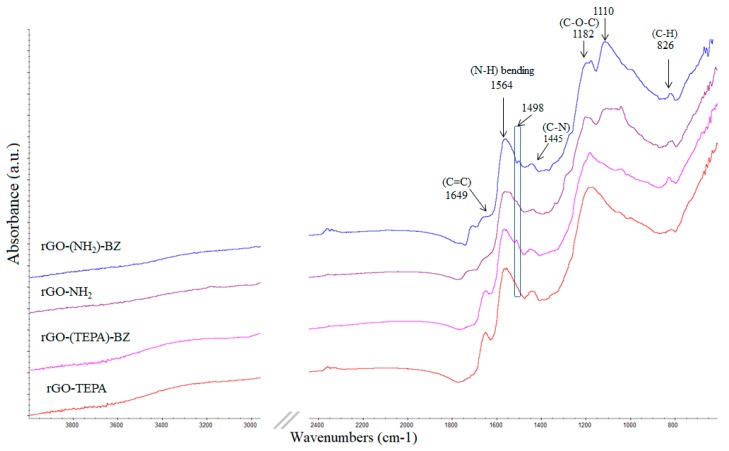
FT-IR spectra of raw materials and benzoxazine-functionalized rGO hybrid monomers.

As depicted in Figure 1, the raw materials showed the characteristic peaks of reduced graphene oxide and amino groups. The signal from 1649 cm^−1^ (C=C) was attributed to the skeletal vibrations of the graphene oxide sheets. As expected, nitrogen functional groups were registered in both rGO–TEPA and rGO–NH2 spectra. Signals from 1546 and 1445 cm^−1^ were due to the N–H bending and C–N stretching vibrations from TETA and amino-PEG structures from rGO surface. In the case of rGO–NH_2_, a new signal may be observed at 1182 cm^−1^, which would be attributed to the C–O–C group from PEG structure. The presence of residual oxidized groups on the rGO surface was noticed by the presence of the signal from 1110 cm^−1^ corresponding to C–O stretching. After functionalization with benzoxazine monomer, a small signal was observed at 1498 cm^−1^ corresponding to the disubstituted aromatic ring from benzoxazine structure. The signal from 826 cm^−1^ (C–H) was assigned to the out-of-plane hydrogen wagging from both of the amino rGO. Important FT-IR signals characteristic to benzoxazine structure were overlaid with FT-IR signals from the raw materials. Therefore, XPS spectroscopy was further employed in order to prove the benzoxazine ring formation.

**Figure 2:**
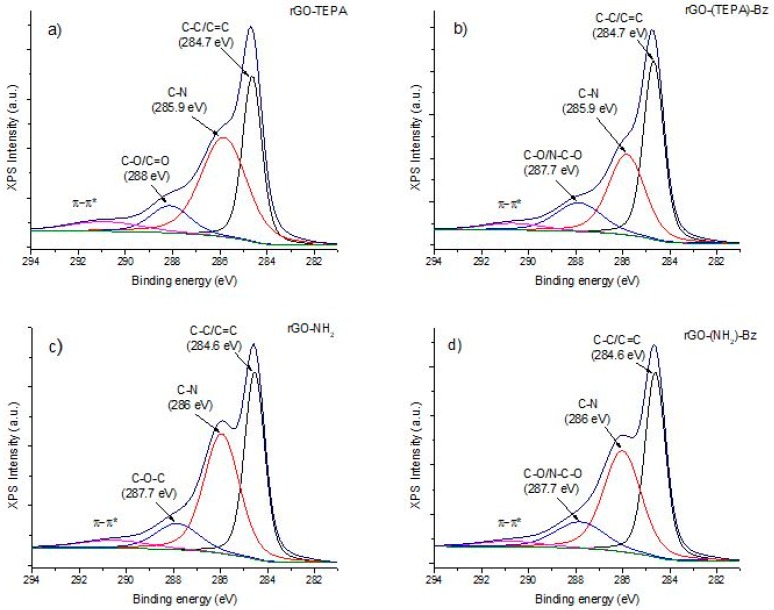
C 1s deconvoluted XPS spectra of (**a**) rGO–TEPA, (**b**) rGO–(TEPA)–BZ, (**c**) rGO–NH_2_, (**d**) rGO–NH_2_–BZ.

XPS was used to identify the surface element states of the investigated materials. C 1s XPS spectra of each sample were deconvoluted into four distinct peaks. As shown in Figure 2, peaks at ~284 eV were assigned to C–C (sp^3^) and C=C (sp^2^) bonds from rGO planar structure. The presence of C–N bonds is noticed at ~286 eV. In case of rGO–TEPA, the appearance of the signal from 288 eV is indicating that C–O/C=O bonds may still be found on the surface of the raw material. The signal at 287.7 eV in the final benzoxazine products may either be due to the C–O bonds present on the surface or due to the new N–C–O groups, which are really a proof of benzoxazine ring formation.

**Figure 3:**
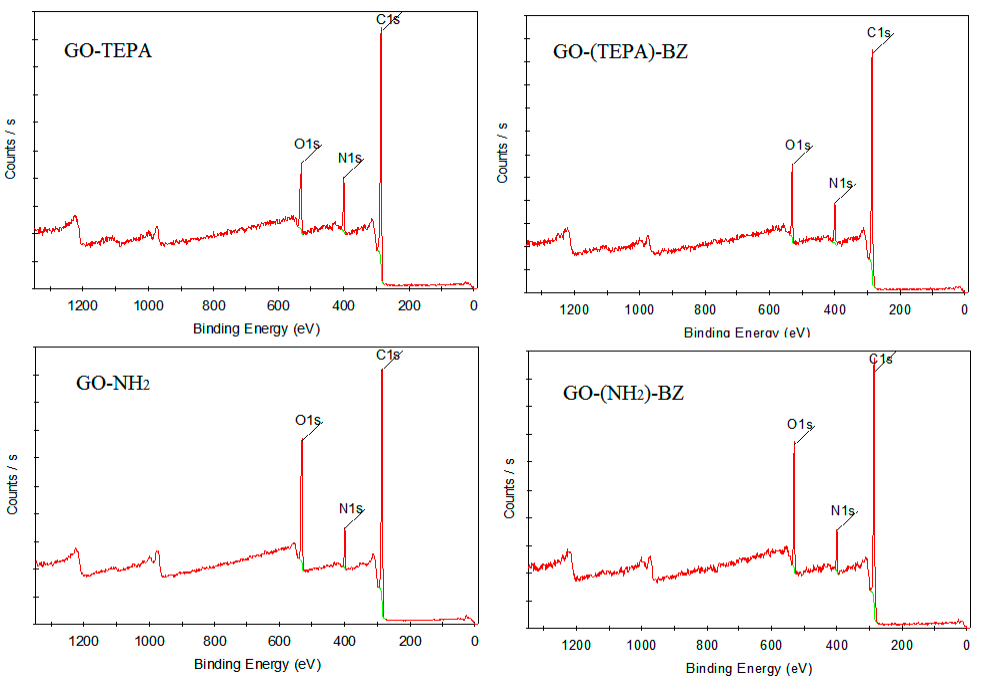
XPS survey spectra of raw materials and benzoxazine-functionalized rGO hybrid monomers.

The XPS survey spectra are also shown in Figure 3 and the corresponding elemental compositions are listed in Table 1. It can be noticed that the N content is lower for the synthesized benzoxazine compounds than for the initial raw materials, since the molar mass increases during the benzoxazine formation and no new N atoms are added into the structures. In addition, it can be observed that the O content is higher for the benzoxazine compounds in comparison to the respective reactants as a high number of oxazine rings are formed, meaning an extra oxygen amount was gained during the reactions.

**Figure 4:**
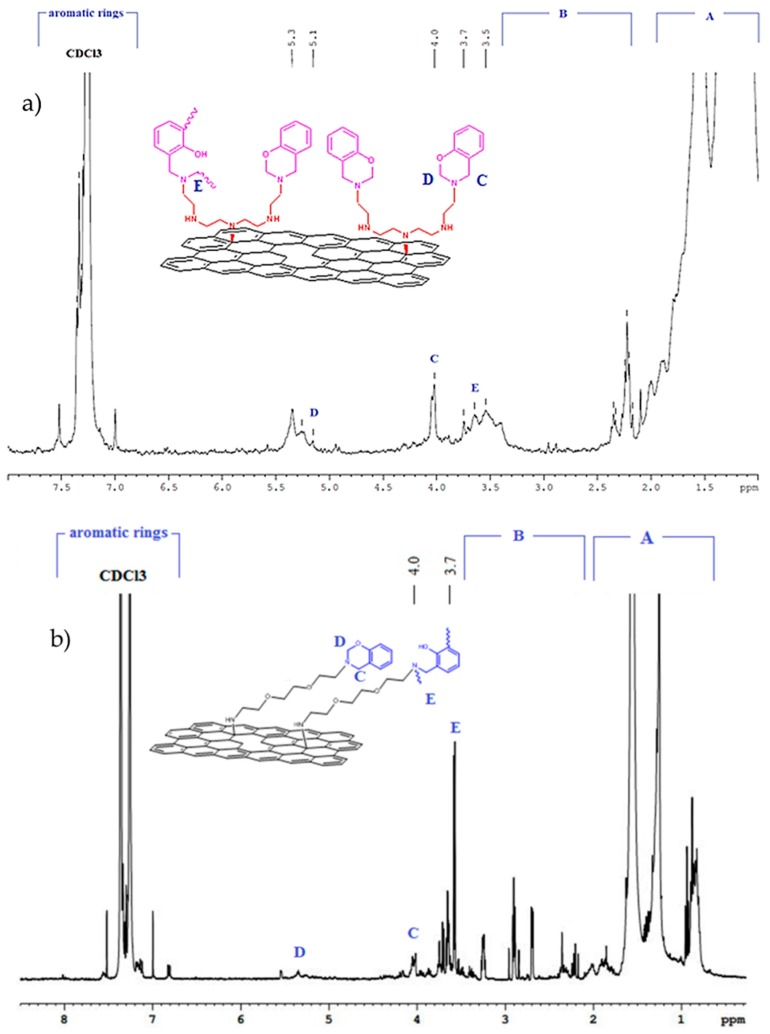
^1^H-NMR spectra of (**a**) rGO–(TEPA) BZ and (**b**) rGO–(NH_2_)–BZ.

The ^1^NMR spectra for the benzoxazine products are shown in Figure 4 and the most significant signals are detailed in Table 2. The most important signals are those denoted as C and D. These were assigned to the protons resulting from the newly formed benzoxazine rings which constitute a significant proof of oxazine rings formation. Additionally, the presence of signal E (at ~3.7 ppm) which was attributed to the protons from the opened benzoxazine structures, indicates that a part of the oxazine rings was quickly opened under the current reaction conditions.

**Figure 5:**
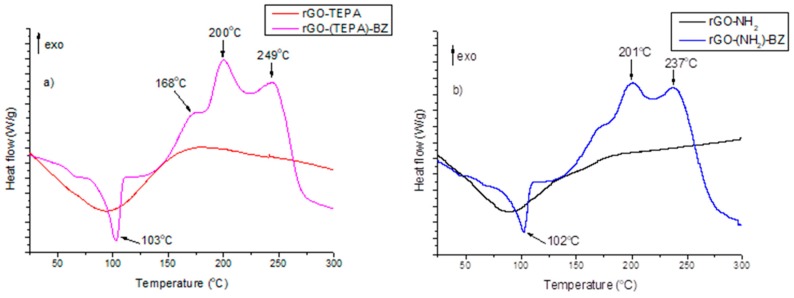
DSC curves of (**a**) rGO–(TEPA)–BZ and (**b**) rGO–(NH_2_)–BZ.

The thermal behaviors of both the rGO–BZ structures were investigated by DSC. Figure 5 reveals the DSC thermograms of the raw materials before and after functionalization with benzoxazine rings. For both rGO–(TEPA)–BZ and rGO–(NH_2_)–BZ a sharp peak around 200 °C can be observed. This corresponds to the ring opening polymerization of the benzoxazine rings. Two other peaks may be denoted from the DSC thermograms, one at ~103 °C corresponding to the decomposition of paraformaldehyde and another one at higher temperatures (230–250 °C) which can be assigned to the degradation process.

**Figure 6:**
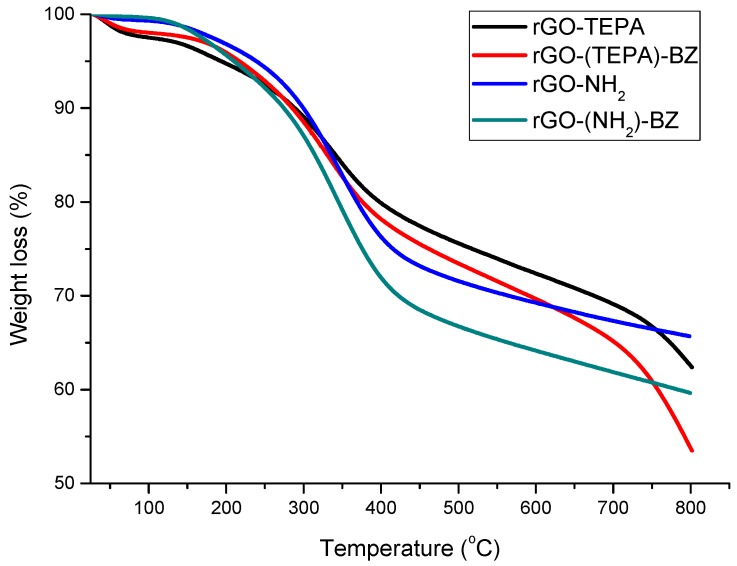
The TGA curves for the raw materials, rGO–(TEPA)–BZ and rGO–(NH_2_)–BZ.

We also studied the thermal behavior of the two samples, using TGA, (as shown in Figure 6) to see if they were modified by the benzoxazine rings. The TGA results showed a higher thermal resistance for rGO–(TEPA)–BZ in comparison to the rGO–TEPA, as the benzoxazine rings obtained onto the rGO surface led to a higher rigidity and thermo-stability. On the contrary, the thermal stability for rGO–(NH_2_)–BZ was lower than that of rGO–NH_2_ due to the fact that a high amount of benzoxazine rings had already polymerized as was noticed from the ^1^H–NMR data (3.7 ppm signal). The weight loss registered for both rGO samples was almost similar, but for the benzoxazine-functionalized rGO samples the weight loss was slightly higher than that of the reactants, since the organic part (BZ) accelerated the thermo-degradation process (Table 3).

**Figure 7:**
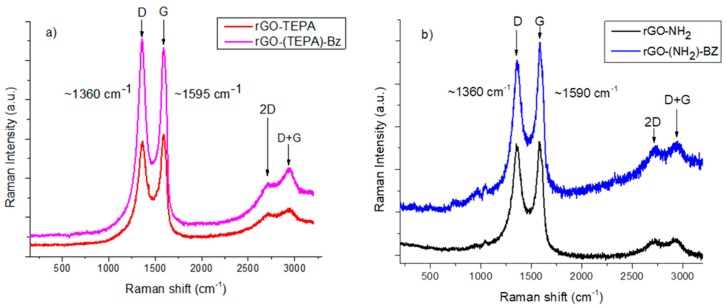
Raman spectra of (**a**) rGO–TEPA and rGO–(TEPA)–BZ, (**b**) rGO–NH_2_ and rGO–NH_2_–BZ.

Raman spectroscopy is a powerful tool for investigating the structural changes in graphene materials because it monitors the D band (~1360 cm^−1^) and G band (~1590 cm^−1^), as is depicted in Figure 7. Each material showed the presence of the D band caused by disordered structure of graphene oxide and the G band which arose from the stretching of C–C bond in graphitic materials being common to all sp^2^ carbon systems. In the case of raw materials, the ratio between the intensity of D band and the intensity of G band (*I*_D_/*I*_G_) was calculated as 0.85 for rGO–TEPA and 0.93 for rGO–NH_2_, respectively (Table 4). This ratio increased after the functionalization with benzoxazine rings in the case of rGO–(TEPA)–BZ, demonstrating that the new defects were created by modification of the rGO surface. In the case of rGO–NH_2_, after functionalization, the *I*_D_/*I*_G_ ratio significantly decreased suggesting a higher tendency for rGO–(NH_2_)–BZ to form an ordered structure. However, in both Raman spectra of benzoxazine-functionalized rGO hybrid monomers two other distinguishable peaks at ~2700 and ~2900 cm^−1^ was observed, which corresponded to the 2D band and (D+G) the combination mod induced by the disorder. A sharper (D+G) combination peak as compared to the raw material, strongly supported that after the functionalization with benzoxazine, the exfoliation of rGO sheets occurred in case of each synthesized material.

**Figure 8:**
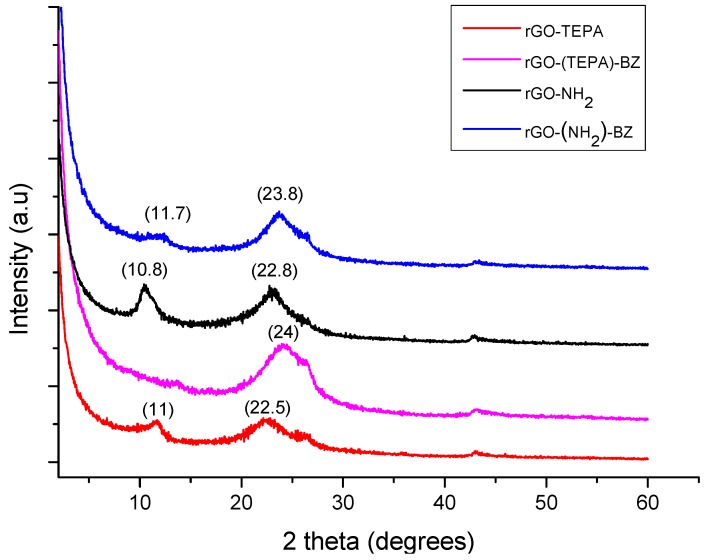
The XRD curves for rGO–(TEPA)–BZ and rGO–(NH_2_)–BZ and raw materials.

The XRD spectra are shown in Figure 8. XRD curves are useful to analyze the structures and the mechanism of exfoliation for the synthesized products. It may be noticed that for rGO–TEPA the signal at 2θ ≅ 11 degrees just disappeared after the synthesis of benzoxazine rings (rGO–(TEPA)–BZ) which means that in this case at least a partially exfoliated structure was formed. On the contrary, for rGO–NH_2_ the signal at 2θ ≅ 11 degrees had significantly decreased in intensity for the corresponding benzoxazine product (rGO–(NH_2_)–BZ), but it is still present which indicates a much lower degree of exfoliation for rGO–(NH_2_)–BZ than for rGO–(TEPA)–BZ.

**Figure 9:**
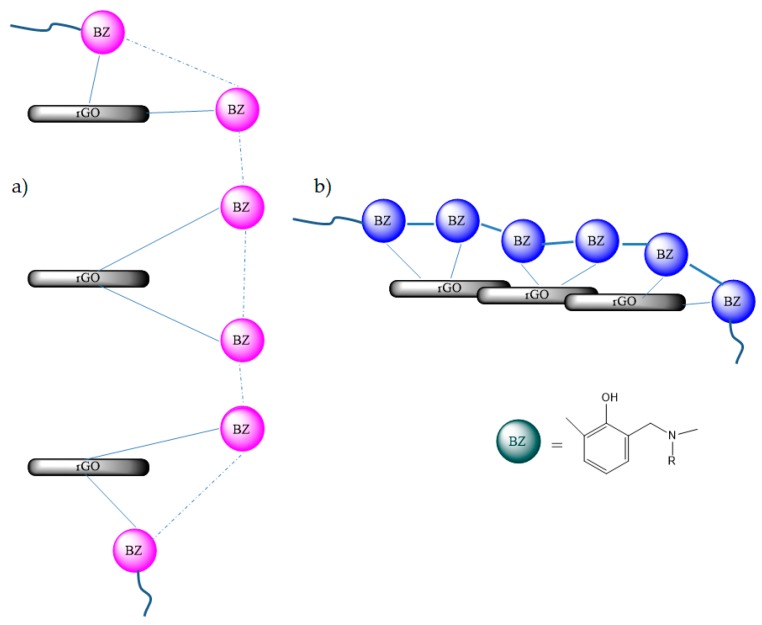
The assembly of benzoxazine products more or less exfoliated caused by more or less bonds formed in between the oxazine rings: (**a**) rGO–(TEPA)–BZ and (**b**) rGO–(NH_2_)–BZ.

The correlation of XRD data and ^1^H-NMR spectra may offer significant details of the final structures of benzoxazine products. Thus in the case of rGO–(NH_2_)–BZ the ^1^H–NMR signal at 3.7 ppm assigned to ring-opening structures was much sharper than for rGO–(TEPA)–BZ meaning that for rGO–(NH_2_)–BZ more opened structures were formed. On the other hand, it was proved from XRD spectra that the rGO–(NH_2_)–BZ exhibited a lower degree of exfoliation than for the rGO–(TEPA)–BZ. Therefore, it may be concluded that as the number of opened benzoxazine rings increases, the exfoliation process is hindered by the bonds formed between the oxazine rings. The exfoliation process is facilitated, in the case of rGO–(NH_2_)–BZ, for which the close position of primary –NH_2_ groups makes it easier to form bonds in the form of a chain of polymer by using the ring-opening process. In the case of rGO–(TEPA)–BZ, the benzoxazine groups are much further away one from another, so the ring-opening process occurs in a different manner. As a result, the graphene sheets may be exfoliated since a polymer chain does not keep them stacked together (Figure 9).

**Figure 10:**
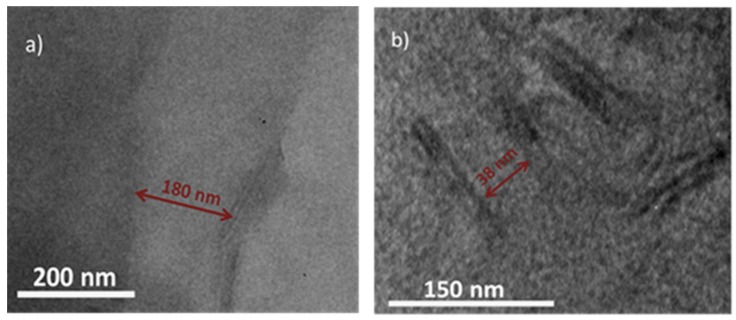
TEM images for: (**a**) rGO–(TEPA)–BZ and (**b**) rGO–(NH_2_)–BZ.

The TEM images (Figure 10) confirm the XRD data and show that the structure of rGO–(TEPA)–BZ is highly intercalated in some zones. The structure even is exfoliated in comparison to rGO–(NH_2_)–BZ which exhibits a more compact structure, and is almost similar to a classical composite material which have the graphene sheets agglomerated into the polymer mass.

## 4. Conclusions

New structures of benzoxazine monomers which were growing directly onto the reduced graphene oxide (rGO) surface, were synthesized from a modified rGO, rGO–TEPA and rGO–NH_2_ respectively. The ^1^H–NMR spectra of benzoxazine structures revealed that oxazine rings were partially polymerized. This was an expected result, as the availability of benzoxazine ring for the ring-opening polymerization is rather high even at moderated temperatures.

The XRD spectra showed a significant difference between the structure of rGO–(TEPA)–BZ and rGO–(NH_2_)–BZ, as the exfoliation process had a higher chance of occurrence in the case of rGO–(TEPA)–BZ than in that of rGO–(NH_2_)–BZ. This conclusion may be clearly explained by looking at the ^1^H–NMR spectra which show the signals from the polymerization of oxazine rings. Thus, the exfoliation process is strongly influenced by the polymerization of oxazine rings formed onto the rGO surface. As the polymerization process gets many chances to occur (in the case of rGO–(NH_2_)–BZ, due to the availability of the oxazine rings which are close to one another), the exfoliation of rGO sheets will be hindered by the strong bonds formed within the polymer. If the benzoxazine rings are have a greater distance between them (as for rGO–(TEPA)–BZ), the polymerization is unlikely to occur and the rGO sheets are freer to separate and form an exfoliated structure. The new benzoxazine-based structures that have been synthesized, in this experiment, are potentially good candidates for the electronic industry. This is because they were produced from rGO based compounds which did not suffer any uncontrolled oxidation that might affect the regular sp^2^ structure, and hence, also affect its electrical properties.

## Figures and Tables

**Table 1:** Elemental composition from XPS data of raw materials and benzoxazine-functionalized rGO hybrid monomers.

Sample	C (%)	O (%)	N (%)
rGO–TEPA	79.1	9.7	11.2
rGO–(TEPA)–BZ	79.3	12.1	8.6
rGO–NH_2_	78.3	14.9	6.8
rGO–(NH_2_)–BZ	78.6	15.3	6.1

**Table 2:** The significant signals in ^1^H-NMR spectra of rGO–(TEPA)–BZ and rGO–(NH_2_)–BZ.

^1^H-NMR Signal Position		^1^H-NMR Signal Assignments
A	1.2–2 ppm	–CH_2_– with no attached functional groups from rGO
B	2–2.8 ppm	protons between C–N from TEPA structure
C	4.0 ppm	protons from Ar–CH_2_–N–
D	5.1 ppm	protons from N–CH_2_–O–
E	3.7 ppm	–CH_2_– from opened benzoxazine ring
	6.7–7.3 ppm	protons from aromatic rings

**Table 3:** The TGA data for the raw materials and benzoxazine-functionalized rGO.

Sample	Td3% (°C)	Td5% (°C)	Weight Loss (%)
rGO–TEPA	137	195	38
rGO–(TEPA)–BZ	173	217	47
rGO–NH_2_	199	242	34
rGO–(NH_2_)–BZ	108	212	40

**Table 4:** Raman data for raw materials and benzoxazine-functionalized rGO hybrid monomers.

Sample	*I*_D_/*I*_G_
rGO–TEPA	0.85
rGO–(TEPA)–BZ	0.99
rGO–NH_2_	0.93
rGO–(NH_2_)–BZ	0.82
